# Methyltransferase METTL8 is required for 3-methylcytosine modification in human mitochondrial tRNAs

**DOI:** 10.1016/j.jbc.2022.101788

**Published:** 2022-03-03

**Authors:** Jenna M. Lentini, Rachel Bargabos, Chen Chen, Dragony Fu

**Affiliations:** Department of Biology, Center for RNA Biology, University of Rochester, Rochester, New York, USA

**Keywords:** METTL8, tRNA, tRNA modification, 3-methylcytosine, m^3^C, mitochondria, m3C, 3-methylcytosine, METTL8, methyltransferase-like 8, mt, mitochondrial, MTS, mitochondrial targeting sequence, ORF, open reading frame, PHA, Positive Hybridization in the Absence of Modification, RFP, red fluorescent protein, RT, reverse transcriptase, sgRNAs, sequence guide RNAs

## Abstract

A subset of eukaryotic tRNAs is methylated in the anticodon loop, forming 3-methylcytosine (m^3^C) modifications. In mammals, the number of tRNAs containing m^3^C modifications has been expanded to include mitochondrial (mt) tRNA-Ser-UGA and mt-tRNA-Thr-UGU. However, whereas the enzymes catalyzing m^3^C formation in nuclear-encoded tRNAs have been identified, the proteins responsible for m^3^C modification in mt-tRNAs are unknown. Here, we show that m^3^C formation in human mt-tRNAs is dependent upon the methyltransferase-Like 8 (METTL8) enzyme. We find that METTL8 is a mitochondria-associated protein that interacts with mitochondrial seryl-tRNA synthetase, as well as with mt-tRNAs containing m^3^C. We demonstrate that human cells deficient in METTL8 exhibit loss of m^3^C modification in mt-tRNAs, but not nuclear-encoded tRNAs. Consistent with the mitochondrial import of METTL8, the formation of m^3^C in METTL8-deficient cells could be rescued by re-expression of WT METTL8, but not by a METTL8 variant lacking the N-terminal mitochondrial localization signal. Notably, we found METTL8-deficiency in human cells causes alterations in the native migration pattern of mt-tRNA-Ser-UGA, suggesting a role for m^3^C in tRNA folding. Altogether, these findings demonstrate that METTL8 is required for m^3^C formation in mt-tRNAs and uncover a potential function for m^3^C modification in mitochondrial tRNA structure.

The introduction of chemical modifications into RNA plays an important role in RNA processing, folding, and function (1). tRNA is one of the most highly modified RNA species, accruing more than 10 modifications per tRNA on average (2, 3, 4, 5). These diverse modifications can influence the folding and stability of tRNA species with subsequent impact on tRNA function in proper decoding and protein synthesis (6, 7, 8).

The human mitochondrial genome encodes 22 tRNA species that are essential for decoding the 13 mRNAs necessary for proper mitochondrial respiration (9, 10). Human mt-tRNAs are transcribed by mitochondrial RNA polymerase and are subsequently modified by nuclear-encoded tRNA modification enzymes that are imported into the mitochondria (11, 12, 13). Anticodon loop modifications in mitochondrial (mt)-tRNA play a critical role in mitochondrial protein synthesis by allowing the 22 mt-tRNAs increased decoding capacity for all 60 codons (14). Moreover, modifications elsewhere in the sequence of mt-tRNAs are necessary for efficient folding and stability (15, 16). Several human diseases are associated with pathogenic variants of mt-tRNA modification enzymes, underscoring the importance of mt-tRNA modifications in tRNA function and cellular physiology (10, 14, 17, 18, 19, 20, 21).

In human cells, mt-tRNA-Ser-UGA and mt-tRNA-Thr-UGU contain the 3-methylcytosine (m^3^C) modification at position 32 of the anticodon loop (Fig. 1) (12, 22, 23). In addition to mt-tRNA-Ser-UGA and mt-tRNA-Thr-UGU, the m^3^C modification is present in a subset of human nuclear-encoded tRNAs (24). The m^3^C modification is hypothesized to influence tRNA folding since the nucleotide at position 32 can form a noncanonical base-pair with residue 38 in certain tRNAs (25, 26, 27). Moreover, loss of m^3^C in tRNA-Ser of mouse stem cells causes alterations in gene expression and ribosome occupancy (28). However, the exact molecular role of m^3^C in tRNAs remains unknown. Moreover, the enzymes responsible for m^3^C formation in mt-tRNAs have not been identified.

**Figure 1:**
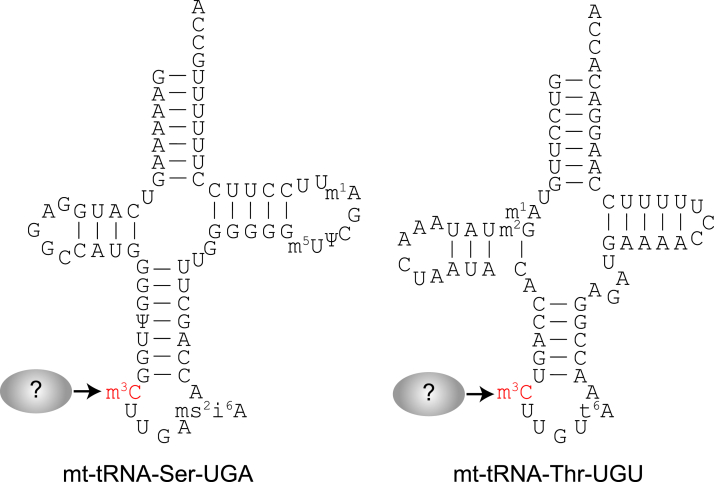
**Secondary structures of human mitochondrial tRNAs containing the m**^**3**^**C modification.** The m^3^C modification in the anticodon loop is shown in *red*. m3C, 3-methylcytosine.

In nuclear-encoded tRNAs, the formation of m^3^C is dependent upon the Trm140p family of methyltransferases (29, 30). While budding yeast *Saccharomyces cerevisiae* encodes a single Trm140 enzyme, fission yeast *Schizosaccharomyces pombe* encodes two Trm140 homologs that are separately responsible for modifying tRNA-Thr or tRNA-Ser isoacceptors (24). Intriguingly, humans encode four Trm140 homologs that include METTL2A, METTL2B, METTL6, and METTL8. METTL2A and METTL2B encode paralogous proteins that share 99% amino acid sequence identity. Knockout of METTL2A/B in human cells abolishes m^3^C formation in a subset of cytoplasmic tRNA-Thr and tRNA-Arg isoacceptors, while METTL6-deficiency in mouse cells leads to loss of m^3^C in cytoplasmic tRNA-Ser species (31). In addition, *S. cerevisiae* Trm140 forms an interaction with seryl-tRNA synthetase, which is required for stimulating the methyltransferase activity of Trm140p on tRNA-Ser isoacceptors (32). We have also shown that human METTL2A and METTL2B interact with a tRNA synthetase mimic that is required for m^3^C formation in tRNA-Arg-CCU and UCU in human cells (33). These studies highlight the evolutionary expansion of Trm140 homologs and the diversification of their RNA substrates through interactions with additional protein factors.

In contrast to METTL2A/B and METTL6, loss of METTL8 expression in human or mouse cells had no detectable impact on m^3^C formation in nuclear-encoded tRNAs (31). Instead, METTL8-deficient mouse and human cells exhibited a reduction in m^3^C modification in poly-A selected RNAs that were depleted of tRNAs and rRNAs. These results suggest a possible role for METTL8 in the formation of m^3^C in mRNAs, but the identity of these RNAs is unknown. In addition, METTL8 forms a nuclear RNA-binding complex that associates with numerous RNAs that could also be targets of methylation, including rRNAs (34). However, a recent study using a newly developed sequencing method identified m^3^C in tRNAs but not in other RNAs above the limit of detection (22). Thus, the exact RNA targets of METTL8 remain equivocal.

Here, we show that METTL8 contains an N-terminal mitochondrial targeting sequence (MTS) that is necessary for association with mitochondria. Moreover, we find that METTL8 interacts with mitochondrial seryl-tRNA synthetase as well as m^3^C-containing mt-tRNAs. Using CRISPR-mediated gene editing, we demonstrate that METTL8-knockout in human cells abolishes m^3^C modification in mt-tRNA-Ser-UGA and mt-tRNA-Thr-UGU. Notably, mt-tRNA-Ser-UGA lacking m^3^C exhibits altered migration on native gels indicative of perturbed folding. In total, these results identify METTL8 as the protein responsible for m^3^C formation in mt-tRNAs and uncover a putative role for m^3^C in maintaining proper tRNA folding.

## Results

### METTL8 harbors an N-terminal mitochondrial localization signal

**Figure 2:**
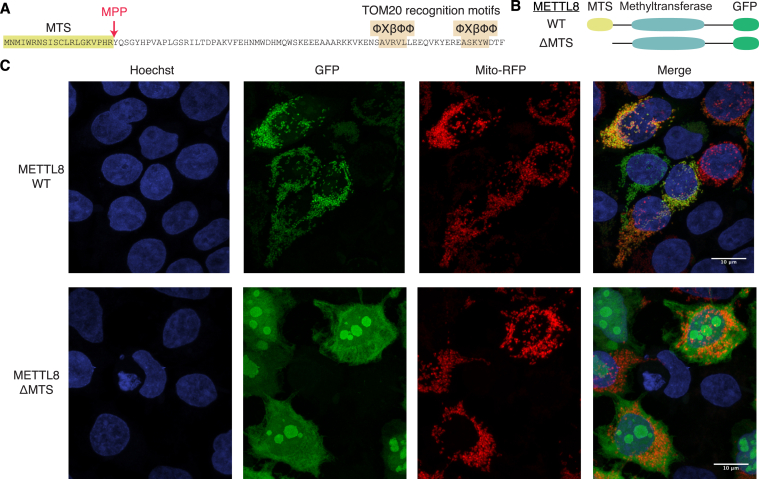
**METTL8 exhibits localization in mitochondria that is dependent upon amitochondrial targeting sequence(MTS)**. *A*, sequence of the N-terminus of METTL8 with predicted MTS, proteolytic cleavage sites, and TOM20 recognition motifs. φXβφφ is the TOM20 recognition motif where φ is a hydrophobic residue, X is any residue, and β is a basic residue. *B*, representative schematic of METTL8 fusion proteins tagged with GFP at the carboxy terminus. WT METTL8 or METTL8 lacking the MTS (ΔMTS) are depicted. *C*, confocal microscopy images of 293T cells transiently transfected with constructs expressing METTL8-WT or METTL8-ΔMTS fusion proteins with GFP. Mitochondria were identified using mitochondrion-targeted red fluorescent protein and nuclear DNA was stained with Hoechst. Overlap of red mitochondria and green GFP signal is displayed by *yellow* merged color. METTL8, methyltransferase-like 8.

To identify proteins responsible for m^3^C formation in mt-tRNAs, we first examined whether any of the four human Trm140 homologs possess a putative MTS. Using predictive algorithms for identifying an N-terminal MTS (35), human METTL6, METTL2A, and METTL2B exhibited less than 3% probability of containing an MTS, whereas METTL8 displayed an 84% probability of containing an MTS (Fig. 2*A*, MTS, highlighted sequence). Moreover, METTL8 was predicted to contain a consensus cleavage site for the mitochondrial processing peptidase as well as sequence recognition motifs for the TOM20 mitochondrial import machinery (Fig. 2*A*, MPP, TOM20). We also noted that METTL8 has been detected in purified mitochondria from human and mouse cells *via* high-throughput proteomic experiments and proximity-labeling (36, 37).

To determine the subcellular localization of METTL8, we transiently transfected 293T human cells with constructs expressing human METTL8 fused at its carboxy terminus to GFP (Fig. 2*B*, WT). We also tested the functional requirement for the predicted MTS in METTL8 by generating a construct expressing METTL8 lacking the MTS signal (Fig. 2*B*, ΔMTS). As a marker for mitochondria, we coexpressed a red fluorescent protein (RFP) fused to the MTS of E1 alpha pyruvate dehydrogenase with the METTL8-GFP fusion proteins. METTL8-GFP exhibited a punctate, fragmented pattern of cytoplasmic localization surrounding the nucleus in human cells (Fig. 2*C*, METTL8-WT, GFP). The fluorescence signal for METTL8 exhibited overlap with the RFP-tagged mitochondrial marker in nearly all cells that expressed both METTL8-GFP and the mitochondrial RFP marker (Fig. 2*C*, Merge, yellow signal, see Fig. S1 for more image fields). In contrast to WT METTL8, METTL8-ΔMTS exhibited diffuse cytoplasmic localization along with enrichment in the nucleolus (Fig. 2*C*, METTL8-ΔMTS, GFP). Compared to the METTL8-GFP subcellular localization pattern, the diffuse cytoplasmic ΔMTS-GFP signal exhibited weak overlap with the RFP mitochondrial maker (Fig. 2*C*, METTL8-ΔMTS, Merge). These findings provide evidence that METTL8 contains a functional MTS sequence that is necessary for proper mitochondrial import. Moreover, loss of the MTS results in the nuclear import of METTL8 and apparent localization into the nucleolus.

### METTL8 forms a complex with nuclear proteins as well as mitochondrial SARS2

**Figure 3:**
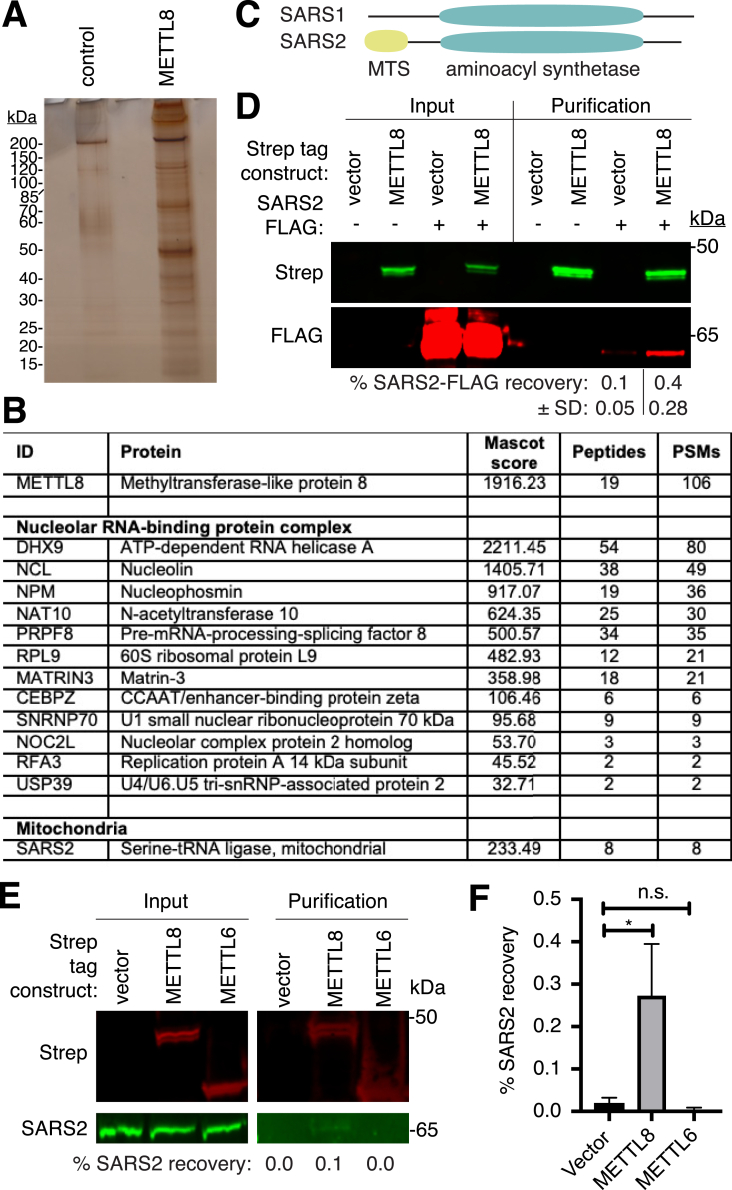
**Identification of METTL8-interacting proteins.***A*, silver stain analysis of Strep-Tactin purifications from human 293T cell lines expressing either vector control or METTL8-Strep. *B*, LC-MS analysis of peptides present in Strep-METTL8 purifications. Table lists a selection of proteins that were enriched in the Strep-METTL8 purification compared to empty vector control. *C*, schematic comparison of SARS1 *versus* SARS2 with aminoacyl synthetase domain and mitochondrial targeting sequence (MTS) indicated. *D*, immunoblot analysis of empty vector control and Strep-METTL8 purifications from transient transfections. The immunoblot was probed with anti-Twin-Strep and anti-FLAG antibodies. The input represents 1% of total. Percent (%) recovery represents the fraction of SARS2-FLAG recovered from the total input in the vector or METTL8 purification. E and F, immunoblot analysis of empty vector control, METTL8, and METTL6 purifications from transient transfections. The immunoblot was probed with anti-Twin-Strep and anti-SARS2 antibodies. Input represents 4% of total extract used for purification. Percent (%) recovery represents the fraction of SARS2 recovered from the total input in the vector, METTL8, and METTL6 purifications. ∗*p* < 0.05. *p* = 0.0232 for vector *versus* METTL8, 0.2089 for vector *versus* METTL6. METTL8, methyltransferase-like 8; PSM, peptide spectral matches.

Previous studies have shown that *S. cerevisiae* Trm140 interacts with seryl-tRNA synthetase, which stimulates the methyltransferase activity of Trm140 with tRNA-Ser isoacceptors (32). Moreover, we have previously shown that human METTL2 forms a complex with the tRNA synthetase mimic DALRD3 to catalyze m^3^C formation in tRNA-Arg-CCU and tRNA-Arg-UCU (33). To gain insight into the protein interaction network of human METTL8, we stably expressed METTL8 fused to the Twin-Strep tag at the C-terminus in 293T human embryonic cells (38). METTL8-Strep was affinity purified from whole cell extracts on Strep-Tactin resin, eluted with biotin, and analyzed by silver staining. As a control for background contaminants, we performed a parallel purification from human cells integrated with an empty vector. Compared to the control, the METTL8 purification yielded a complex profile of interacting proteins ranging in size from 10 to 200 kDa (Fig. 3*A*).

To identify METTL8-interacting proteins, we performed a large-scale purification from human cells expressing control or METTL8-Strep and subjected the entire eluted samples for proteomic analysis by LC-MS. After subtraction of contaminating proteins found in the control purification, we compiled an inventory of proteins that copurified specifically with METTL8-Strep (see Supporting Information). Peptide sequences corresponding to METTL8 were detected in the purification from cells expressing METTL8-Strep, confirming the successful isolation of Strep-tagged METTL8 (Fig. 3*B*, METTL8). Consistent with a previous study (39), we found that many METTL8-interacting proteins are part of a large nuclear complex enriched for proteins involved in RNA binding and R-loop regulation (Fig. 3*B*, Nucleolar RNA-binding protein complex). Since the function of this nuclear METTL8 complex has been characterized, we focused our subsequent efforts on novel METTL8-interacting proteins.

In addition to nuclear proteins, we detected numerous peptides for mitochondrial seryl-tRNA synthetase (SARS2) in the METTL8 purification that were not found in the control purification (Fig. 3*B*, SARS2). SARS2 shares a homologous aminoacyl synthetase domain as the cytoplasmic seryl-tRNA synthetase SARS1 but also contains an N-terminal MTS (Fig. 3*C*). No other tRNA synthetases were specifically found in the METTL8 purification. To confirm the interaction between METTL8 and mitochondrial SARS2, we transiently expressed METTL8-Strep with FLAG-tagged SARS2. Following purification of METTL8-Strep on Strep-Tactin resin, we monitored the copurification of SARS2-FLAG. Immunoblot analysis revealed the enrichment of SARS2-FLAG with Strep-METTL8 above the background in the vector control purification (Fig. 3*D*). We also validated the METTL8-SARS2 interaction by probing for endogenous SARS2 in purified METTL8-Strep samples. In addition, we tested if SARS2 could interact with METTL6, a different Trm140 homolog. Using this approach, we detected the copurification of endogenous SARS2 with METTL8 that was not readily apparent with the control or Strep-METTL6 purifications (Fig. 3, *E* and *F*). We noted that SARS2 was not identified in a previous study on METTL8-interacting proteins, most likely due to the use of an N-terminal tag that would perturb localization of METTL8 to mitochondria (39). In contrast, we used a carboxy-terminal tag on METTL8, which would not disrupt recognition of the N-terminal MTS by the mitochondrial import machinery as shown with METTL8-GFP above. The identification of mitochondrial SARS2 as a METTL8-interacting protein suggests that METTL8 could play a role in modifying mitochondrial RNAs such as mt-tRNA-Ser-UGA, which contains m^3^C.

### METTL8 interacts with mt-tRNAs

**Figure 4:**
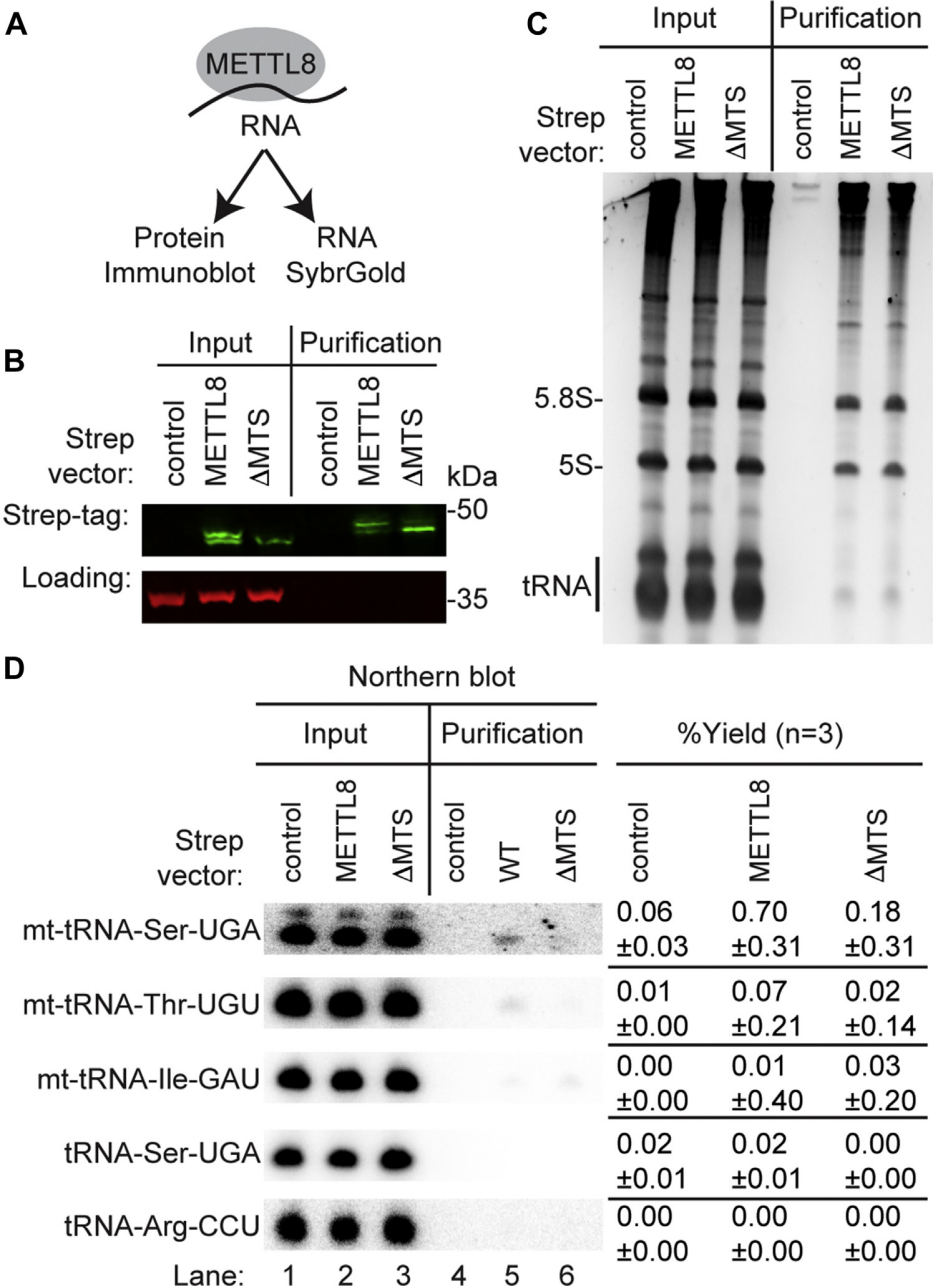
**METTL8 interacts with mt-tRNA-Ser-UGA and mt-tRNA-Thr-UGU.***A*, purification of METTL8 and analysis of protein and RNA. *B*, immunoblot analysis of Strep-Tactin purifications from human cells expressing control, METTL8, or METTL8-ΔMTS fused to the twin-Strep tag. The immunoblot was probed with anti-TwinStrep and anti-actin antibodies. *C*, nucleic acid stain of RNAs extracted from the indicated input or purified samples after denaturing PAGE. The migration pattern of 5.8S rRNA (∼150 nt), 5S rRNA (∼120 nt), and tRNAs (∼70–80 nt) are denoted. *D*, Northern blot analysis of the gel in (*C*) using the indicated probes. Input represents 2% of total extracts used for purification. The percentage yield represents the amount of RNA in the Strep purification that was recovered from the total input. The experiment was repeated three times with comparable results. METTL8, methyltransferase-like 8; mt, mitochondrial.

Based on the localization of METTL8 in mitochondria along with its interaction with mitochondrial SARS2, we next tested whether METTL8 displays stable interactions with mt-tRNAs containing m^3^C. For these experiments, we transiently transfected 293T human cells with constructs expressing either METTL8-WT or METTL8-ΔMTS fused to the Strep tag. After purification on Strep-Tactin resin, a portion of each purification was retained for the confirmation of protein isolation while the remainder was processed for RNA extraction (Fig. 4*A*). Using immunoblotting, we confirmed the expression and purification of METTL8-WT or METTL8-ΔMTS on Strep-Tactin resin (Fig. 4*B*). Both the METTL8-WT or METTL8-ΔMTS purifications exhibited an enrichment of 5S and 5.8S rRNA along with high molecular weight RNA species (Fig. 4*C*). The purification of rRNA with METTL8 is consistent with the copurification of ribosomal proteins along with the localization of METTL8 at R-loops in nucleolar rDNA genes (34). We note that METTL8-ΔMTS exhibits enriched localization in the nucleolus (Fig. 2), which could promote nonspecific interaction with rRNA and tRNA leading to the similar amounts of RNA copurifying with METTL8-WT and METTL8-ΔMTS.

In addition to rRNA, we detected an RNA band migrating at the length of tRNAs that copurified with both METTL8 and METTL8-ΔMTS (Fig. 4*C*). Based upon the mitochondrial localization of METTL8, we hypothesized that the tRNAs copurifying with METTL8 were the two mt-tRNAs containing m^3^C; mt-tRNA-Ser-UGA and mt-tRNA-Thr-UGU. Using Northern blot probing, we found the METTL8 purification was enriched for mt-tRNA-Ser-UGA and mt-tRNA-Thr-UGU above the level of background binding in the control purification (Fig. 4*D*, METTL8-WT). The enrichment of mt-tRNA-Ser-UGA and mt-tRNA-Thr-UGU was reduced in the METTL8-ΔMTS purification, consistent with the requirement for the MTS in METTL8 mitochondrial localization (Fig. 4*D*, METTL8-ΔMTS). Moreover, neither METTL8 nor METTL8-ΔMTS exhibited enrichment above background for mt-tRNA-Ile, which lacks m^3^C (Fig. 4*D*). In addition, no detectable enrichment was found for nuclear-encoded tRNAs containing m^3^C such as tRNA-Ser-UGA or tRNA-Arg-CCU in either METTL8 purification. The enrichment of mt-tRNA-Ser-UGA and mt-tRNA-Thr-UGU with METTL8 was repeated in an independent METTL8 purification with comparable results (Fig. S2). These results provide evidence that a subpopulation of METTL8 is imported into mitochondria where it interacts with mt-tRNAs containing the m^3^C modification.

### METTL8 is required for m^3^C formation in mt-tRNAs

**Figure 5:**
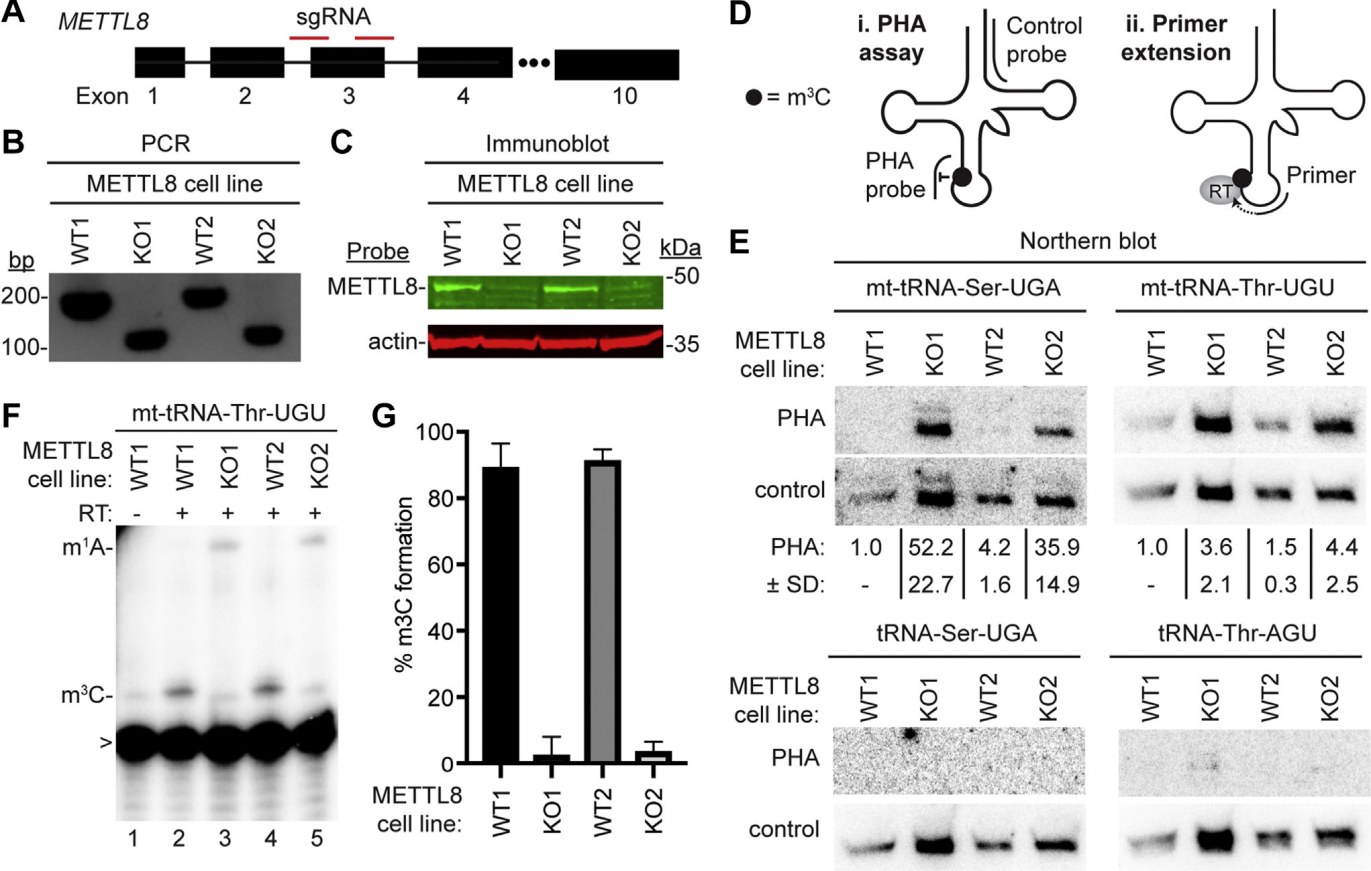
**METTL8 is required for efficient m**^**3**^**C formation in mitochondrial tRNAs *in vivo*.***A*, CRISPR/Cas9 gene KO strategy depicting sequence guide RNAs (sgRNAs) targeting exon 3 of the human *METTL8* gene. *B*, genomic PCR demonstrating the loss of exon 3 in METTL8-KO cell lines. Deletion of exon 3 results in a 72 base-pair deletion. *C*, immunoblot analysis of METTL8 expression in WT *versus* METTL8-KO human cell lines. Actin was used as a loading control. *D*, (i) schematic of the Positive Hybridization in the Absence of Modification (PHA) to monitor m^3^C status. PHA probe spans position C32 while the control probe spans a region lacking modifications on the same tRNA. (ii) schematic of primer extension assay to monitor m^3^C modification. *E*, Northern blot analysis using the PHA assay with probes designed to detect m^3^C at position 32 and a control probe that hybridizes to a different area of the same tRNA. PHA quantification represents the ratio of PHA *versus* control probe signal expressed relative to the WT1 cell line. Northern blotting and quantification were performed on three independent cell preparations for each cell line with comparable results. *F*, representative gel from primer extension assays to monitor the presence of m^3^C in mt-tRNA-Thr-UGU from the indicated cell lines. *G*, quantification of m^3^C formation in (*F*). Primer extension analysis was repeated three times and bars represent the standard deviation from the mean. METTL8, methyltransferase-like 8; mt, mitochondrial; m^1^A, 1-methyladenosine; m^3^C, 3-methylcytosine; RT, reverse transcriptase; >, labeled oligonucleotide used for primer extension; .

The mitochondrial localization of METTL8 along with the copurification of mt-tRNA-Ser-UGA and mt-tRNA-Thr-UGU with METTL8 suggests that METTL8 could play a role in m^3^C formation in mt-tRNAs. To investigate the functional role of METTL8, we generated human METTL8-KO cell lines by CRISPR/Cas9 gene editing (Fig. 5*A*). Using the HAP1 human haploid cell line, we generated two METTL8-KO cell clones containing a 72 base-pair deletion in exon 3 of the *METTL8* gene that was confirmed by PCR and Sanger sequencing (Fig. 5*B*). The deletion is predicted to cause a translation frameshift that results in nonsense-mediated decay or production of a truncated METTL8 missing most of the polypeptide. Using immunoblotting, we detected a substantial reduction of full-length METTL8 protein in both METTL8-KO cell lines compared to the isogenic WT control cell lines (Fig. 5*C*). No major change in cellular proliferation or mitochondrial membrane potential was detected between the isogenic WT or METTL8-KO cell lines (Fig. S3).

To monitor m^3^C formation in tRNA, we used the Positive Hybridization in the Absence of modification (PHA) assay (40, 41, 42, 43). This Northern blot-based assay relies on differential probe hybridization to tRNA caused by the presence or absence of m^3^C, which impairs base-pairing. Thus, a decrease in m^3^C modification leads to an increase in PHA probe signal that can be normalized against the probe signal from a different region of the same tRNA as an internal control (Fig. 5*D*i). For mt-tRNA-Ser-UGA and mt-tRNA-Thr-UGU, there was a considerable increase in PHA probe signal in the METTL8-KO cell lines compared to WT, indicating the loss of m^3^C modification in these particular tRNAs (Fig. 5*E*, mt-tRNA-Ser-UGA and mt-tRNA-Thr-UGU). The increase in PHA signal for mt-tRNA-Ser-UGA and mt-tRNA-Thr-UGU in the METTL8-KO cell lines was observed in independent probing experiments (Fig. S4). In contrast, no change in PHA signal was detected for nuclear-encoded tRNAs containing m^3^C in the METTL8-KO *versus* control cell lines (Fig. 5*E*, tRNA-Ser-UGA and tRNA-Thr-AGU). We also note that the steady-state levels of all tested tRNAs were similar between the WT and METTL8-KO cell lines based upon the blot signal from the control probe (Fig. 5*E*).

As additional validation, we used a primer extension assay in which the presence of m^3^C blocks extension by a reverse transcriptase (RT), while the lack of m^3^C allows for readthrough and generation of an extended product (Fig. 5*D*ii). We were unable to detect an RT extension product for mt-tRNA-Ser-UGA despite numerous attempts with different primers. This is most likely due to the 2-methylthio-N6-isopentenyladenosine modification present in the anticodon loop that can block primer binding (Fig. 1). However, we did observe an RT stop indicative of m^3^C at position 32 in mt-tRNA-Thr-UGU of both WT cell lines that was absent when RT was not included (Fig. 5*F*, compare lane 1 to lanes 2 and 4). Notably, the m^3^C block in mt-tRNA-Thr-UGU was greatly reduced in both METTL8-KO cell lines with subsequent readthrough to the next RT-blocking modification (Fig. 5*F*, compare lanes 2 and 4 to lanes 3 and 5; quantified in 5G). Combined with the PHA assay results, these findings suggest that METTL8 is required for the formation of m^3^C at position 32 of mt-tRNA-Ser and mt-tRNA-Thr but not the other m^3^C-containing tRNAs encoded by the nuclear genome.

**Figure 6:**
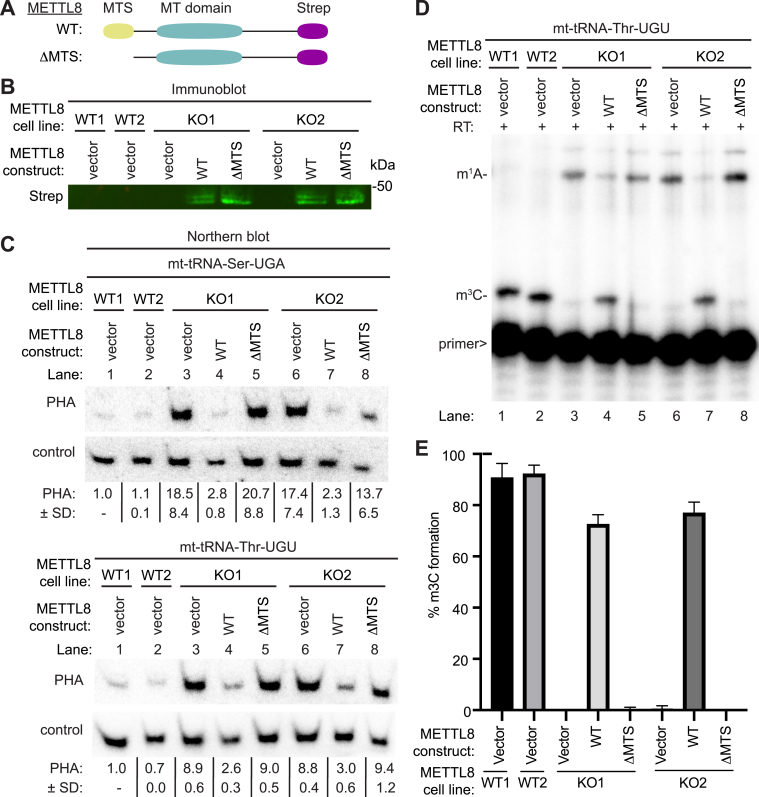
**The MTS of METTL8 is required for efficient rescue of m**^**3**^**C formation in mitochondrial tRNAs of METTL8-KO human cells.***A*, schematic of METTL8 variants used for METTL8 rescue experiments. *B*, immunoblot analysis of Strep-Tactin purifications from the indicated cell lines with anti-Strep antibodies. *C*, Northern blot analysis using the Positive Hybridization in the Absence of Modification (PHA) assay with probes designed to detect m^3^C at position 32 and a control probe that hybridizes to a different area of the same tRNA. PHA quantification represents the ratio of PHA signal to control probe normalized to the WT1 cell line. (*C*) was repeated three times with similar results. *D*, representative gel of primer extension assays to monitor the presence of m^3^C in mt-tRNA-Thr-UGU from the indicated cell lines. *E*, quantification of m^3^C formation in (*D*). Primer extension analysis was repeated three times and bars represent the SD from the mean. m^1^A, 1-methyladenosine; m^3^C, 3-methylcytosine; METTL8, methyltransferase-like 8; MTS, mitochondrial targeting sequence; RT, reverse transcriptase; >, labeled oligonucleotide used for primer extension.

To confirm that loss of m^3^C in mt-tRNAs is due to METTL8-deficiency, we tested whether re-expression of METTL8 in the METTL8-KO cell lines could rescue m^3^C formation in mt-tRNAs. In addition, we tested whether mitochondrial localization of METTL8 is necessary for m^3^C formation in mt-tRNAs using the METTL8-ΔMTS variant. We generated METTL8-KO cell lines containing an integrated lentiviral construct encoding the cDNAs for METTL8 or METTL8-ΔMTS fused to the Strep tag (Fig. 6*A*). Due to the low expression level of the integrated METTL8 transgene in HAP1 human cells, we were unable to detect the expression of METTL8 in cell lysates despite multiple attempts using the anti-METTL8 antibody (Fig. S5). As an alternative, we enriched for the METTL8-Strep fusion protein from cell lysates *via* purification on Strep-Tactin resin followed by immunoblotting with an anti-Strep tag antibody. Using this approach, the expression of either Strep-tagged METTL8-WT or METTL8-ΔMTS could be detected in both METTL8-KO cell lines (Fig. 6*B*).

We next analyzed m^3^C status in the METTL8-KO cell lines expressing METTL8 or METTL8-ΔMTS. As expected, METTL8-KO cells with vector alone exhibited an increased PHA signal for mt-tRNA-Ser-UGA and mt-tRNA-Thr-UGU compared to WT cells indicative of m^3^C-deficiency (Fig. 6*C*, compare lanes 1 and 2–3 and 6). Re-expression of METTL8-WT in either METTL8-KO cell line resulted in a reduced PHA signal for mt-tRNA-Ser-UGA and mt-tRNA-Thr-UGU compared to that of METTL8-KO cells with vector alone (Fig. 6*C*, compare lanes 3 and 6 to lanes 4 and 7). The METTL8-KO cells re-expressing METTL8 exhibited comparable PHA signal for mt-tRNA-Ser-UGA and mt-tRNA-Thr-UGU to that of WT cells (Fig. 6*C*, compare lanes 1 and 2 to lanes 4 and 7). In contrast to WT METTL8, expression of METTL8-ΔMTS had no major change in the PHA signal of mt-tRNA-Ser-UGA or mt-tRNA-Thr-UGU in the METTL8-KO cell lines compared to vector alone (Fig. 6*C*, compare lanes 3 and 6 to lanes 5 and 8).

We also monitored m^3^C formation in the METTL8-rescue cell lines using the primer extension assay described above. As expected, METTL8-KO cell lines with vector alone exhibited no m^3^C stop at position 32 in mt-tRNA-Thr-UGU when compared to either WT cell line (Fig. 6*D*, compare lanes 1 and 2–3 and 6; quantified in 6E). Consistent with the PHA results, we detected restoration of m^3^C modification in mt-tRNA-Thr-UGU of METTL8-KO cells re-expressing METTL8-WT but not METTL8-ΔMTS (Fig. 6*D*, compare lanes 4 and 7 to lanes 5 and 8; quantified in 6E). Overall, these results indicate that re-expression of METTL8 in METTL8-KO cells is sufficient to rescue m^3^C formation in mt-tRNAs, and that mitochondrial localization is required for METTL8 function in m^3^C modification.

### Purified METTL8 exhibits methyltransferase activity on tRNA-Thr-UGU

**Figure 7:**
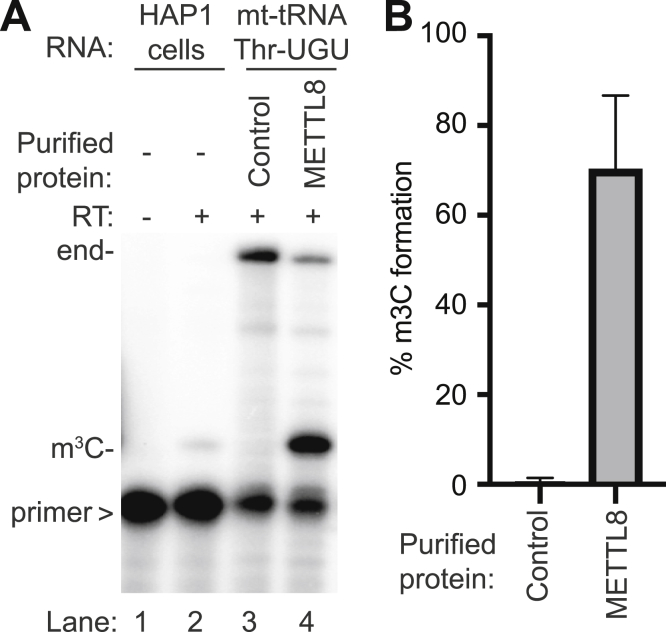
***In vitro* reconstitution of METTL8 methyltransferase activity on mt-tRNA-Thr-UGU.** Purified METTL8 catalyzes m^3^C formation on mt-tRNA-Thr-UGU *in vitro*. *A*, representative gel of primer extension assay to monitor the presence of m^3^C in mt-tRNA-Thr-UGU after incubation with either control or purified METTL8. *B*, quantification of m^3^C formation in (A). Primer extension analysis was repeated three times and error bars represent the SD from the mean. m^3^C, 3-methylcytosine; METTL8, methyltransferase-like 8; mt, mitochondrial; RT, reverse transcriptase; >, labeled oligonucleotide used for primer extension.

Our results suggest that METTL8 is the methyltransferase responsible for m^3^C formation in specific mt-tRNAs. To test METTL8 for enzymatic activity, we expressed and purified METTL8 followed by incubation with *in vitro* transcribed mt-tRNA-Thr-UGU and *S-*adenosylmethionine as the methyl donor. The formation of m^3^C was then monitored by primer extension as described above. As a positive control, we performed primer extension on RNA harvested from WT human HAP1 cells. Using cellular RNA, we detected an RT stop at position 32 of tRNA-Thr-UGU indicative of the m^3^C modification that was absent when no RT was added (Fig. 7*A*, lanes 1 and 2). Notably, we also observed a stop at position 32 of *in vitro* transcribed mt-tRNA-Thr-UGU after incubation with purified METTL8 but not with a control purification (Fig. 7*A*, lanes 3 and 4, quantified in Fig. 7*B*). These results provide evidence that METTL8 can catalyze methylation of mt-tRNA-Thr-UGU at position 32.

### Testing the role of SARS2 in m^3^C formation

Previous studies have shown that *S. cerevisiae* Trm140p and human METTL2A/2B require accessory proteins to efficiently catalyze m^3^C formation in their tRNA substrates. *S. cerevisiae* Trm140p interacts with seryl-tRNA synthetase to catalyze m^3^C formation on tRNA-Ser isoacceptors (32). Human METTL2A/B has evolved an interaction with the arginyl synthetase-like mimic DALRD3 to recognize and methylate tRNA-Arg-CCU and Arg-UCU substrates (33). Since we detected an interaction between METTL8 and SARS2, we investigated whether SARS2 is required for m^3^C formation in human cells. Using CRISPR interference to repress SARS2 gene expression (44), we generated two independent SARS2-knockdown cell lines with reduced SARS2 levels (Fig. S6*A*, quantified in Fig. S6*B*). Based upon the PHA assay, we did not detect a significant change in m^3^C formation on mt-tRNA-Ser-UGA in either SARS2-knockdown cell line compared to control cells (Fig. S6*C*, quantified in Fig. S6*D*). While there was no detectable difference in m^3^C status between cell lines, we note that the reduction of SARS2 to levels that affect m^3^C formation might be unfeasible since SARS2 depletion is known to reduce cell viability (45). Thus, the PHA assay might lack the sensitivity to detect subtle changes in m^3^C levels caused by incomplete depletion of SARS2.

### m^3^C impacts the conformation of mitochondrial tRNA-Ser-UGA

**Figure 8:**
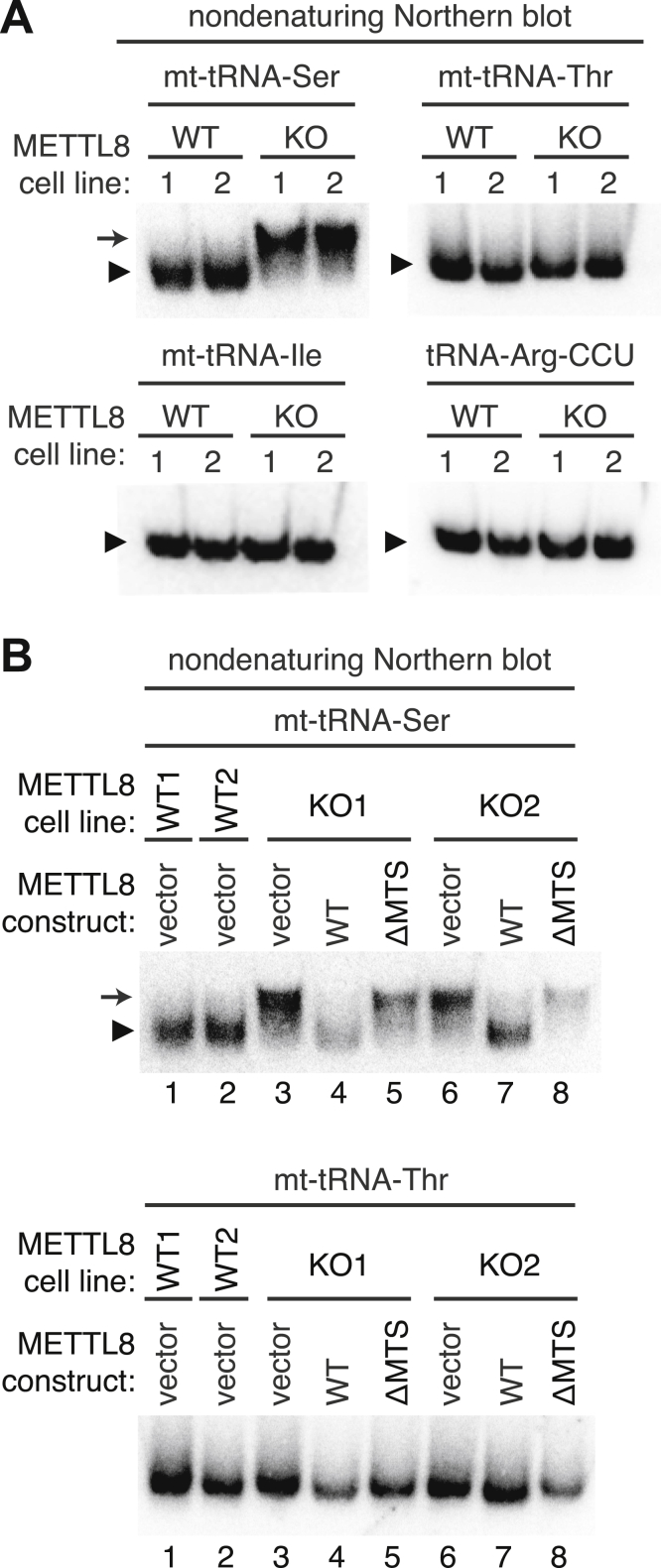
**The native migration pattern of mt-tRNA-Ser is altered in METTL8-KO cell lines.***A*, total RNA from the indicated cell lines was fractionated on nondenaturing gels followed by transfer and hybridization with probes against the indicated tRNAs. The predominant band for each tRNA species in WT control cells is denoted by the *arrowhead*. The slower migrating mt-tRNA-Ser species found in METTL8-KO cell lines is denoted by the *arrow*. *B*, native gel analysis of mt-tRNA-Ser and Thr from the indicated cell lines. The predominant band for each tRNA species is denoted by the *arrowhead* and the slower migrating mt-tRNA-Ser species is denoted by the *arrow*. METTL8, methyltransferase-like 8; mt, mitochondrial.

The precise function of m^3^C modification in tRNA remains enigmatic. However, biophysical modeling studies suggest that the m^3^C modification could impact tRNA structure (46). To investigate a role for m^3^C in the folding of mt-tRNA-Ser-UGA and mt-tRNA-Thr-UGU, total RNA from WT or METTL8-KO human cells was resolved on nondenaturing native polyacrylamide gels followed by Northern blotting (47). Both mt-tRNA-Ser-UGA and mt-tRNA-Thr-UGU from WT human cells migrated predominantly as a single band on native gels (Fig. 8*A*, arrowhead). In contrast, we found that mt-tRNA-Ser-UGA from both METTL8-KO cell lines exhibited a shift to a slower-migrating species (Fig. 8*A*, arrow). The slower-migrating mt-tRNA-Ser-UGA species in the METTL8-KO cell lines was detected in multiple independent native gel experiments (Fig. S7). The change in migration of mt-tRNA-Ser on native gels is likely due to conformation rather than length since no change in migration for mt-tRNA-Ser was detected on denaturing gels between WT *versus* METTL8-KO cells (Fig. 5*E*). Interestingly, no change in migration pattern was observed for mt-tRNA-Thr-UGU between WT *versus* METTL8-KO cell lines even though it also contains m^3^C dependent upon METTL8 (Fig. 8*A*). We also observed no detectable change in the migration of mitochondrial tRNA-Ile-GAU or cytoplasmic tRNA-Arg-CCU between WT *versus* METTL8-KO cell lines.

To investigate the requirement for METTL8 in mt-tRNA-Ser structure, we examined native tRNA structure in the METTL8 cell lines expressing WT METTL8 or METTL8-ΔMTS. In METTL8-KO cell lines with vector alone, mt-tRNA-Ser-UGA existed predominantly as the slower migrating species compared to the faster-migrating species found in WT human cells with vector alone (Fig. 8*B*, compare lanes 1 and 2 to lanes 3 and 6). Importantly, re-expression of METTL8 in the M8-KO cell line returned the migration pattern of mt-tRNA-Ser-UGA back to that of WT human cells consisting of predominantly the faster migrating species (Fig. 8*B*, mt-tRNA-Ser, compare lanes 1 and 2 to lanes 4 and 7). In contrast, expression of METTL8-ΔMTS lacking mitochondrial localization was unable to restore the WT migration pattern of mt-tRNA-Ser-UGA (Fig. 8*B*, compare lanes 1 and 2 to lanes 5 and 8). The mt-tRNA-Ser-UGA of METTL8-KO cell lines expressing METTL8-ΔMTS appeared more similar to the METTL8-KO cell lines with vector alone (Fig. 8*B*, compare lanes 3 and 6 to lanes 5 and 8). Similar to the results above, METTL8-KO cell lines exhibited no detectable change in the migration pattern of mt-tRNA-Thr-UGU regardless of the integrated vector (Fig. 8*B*, tRNA-Thr-UGU). Altogether, these findings suggest that m^3^C plays a role in mt-tRNA-Ser-UGA folding and conformation that is dependent upon the localization of METTL8 in mitochondria.

## Discussion

Here, we provide evidence that METTL8 is a nuclear-encoded tRNA modification enzyme responsible for catalyzing m^3^C formation in mt-tRNA-Ser-UGA and mt-tRNA-Thr-UGU. In addition, we show that METTL8 localization to the mitochondria is required for the efficient formation of m^3^C in mt-tRNAs. Intriguingly, we find that METTL8-deficiency in human cells alters the mobility of mt-tRNA-Ser-UGA on native gels. To our knowledge, this provides the first experimental demonstration that m^3^C may impact the folding of an endogenous cellular tRNA.

mt-tRNAs fold into noncanonical tertiary structures that are inherently less stable (48). In particular, mt-tRNA-Ser-UGA exhibits a variant secondary structure with an extended anticodon stem that is hypothesized to maintain tertiary structure by compensating for additional changes elsewhere (49, 50). Thus, m^3^C may also play an important role in facilitating the proper folding of the anticodon loop that impacts the overall tertiary structure of mt-tRNA-Ser-UGA. The results presented here suggest that mt-tRNA-Ser-UGA could exist in two different conformational states with m^3^C promoting the structure migrating as the slower-migrating form on native gels. The altered migration pattern of mt-tRNA-Ser-UGA could also be due to changes in modifications elsewhere in the tRNA that affects the overall conformation or charge state. The m^3^C modification could also play a role in the folding of mt-tRNA-Thr-UGU that might not be detectable using the native gel system. Future assays using structural probing could reveal the precise conformational differences observed between mt-tRNAs with or without the m^3^C modification.

Deletion of Trm140p in combination with Trm1p in *S. cerevisiae* leads to a slight growth sensitivity to the translation inhibitor, cycloheximide (29). The lack of tRNA modifications catalyzed by Trm140 and Trm1 may impair the proper folding of tRNA substrates and their function in translation, thus rendering the yeast strain sensitive to perturbations in protein synthesis. While no major changes in the cytoplasmic polysome distribution profile was detected in METTL8-deficient human cells (51), our studies suggest that METTL8-catalyzed tRNA modification is likely to impact mitochondrial translation instead. Thus, it will be pertinent to assay for the possible effects of m^3^C modification on mitochondrial protein synthesis in the future.

Using proteomics of purified METTL8 complexes, we have identified a novel interaction between METTL8 and mitochondrial seryl-tRNA synthetase (SARS2). This METTL8-SARS2 complex is analogous to the interaction previously found between *S. cerevisiae* Trm140 with seryl-tRNA synthetase and human METTL6 with cytoplasmic SARS1 (31, 32). Whereas seryl-tRNA synthetase has been shown to greatly stimulate the methyltransferase activity of Trm140, it is unknown whether human SARS1 or SARS2 are necessary for the methyltransferase activity of METTL6 or METTL8, respectively. While the role of SARS2 in METTL8 activity remains equivocal, we hypothesize that SARS2 interacts with METTL8 to aid in the recognition of specific mitochondrial tRNA substrates. In particular, the SARS2 interaction could modulate how METTL8 specifically recognizes mt-tRNA-Ser-UGA but not mt-tRNA-Ser-AGY, which also contains an unmodified C at the equivalent anticodon loop position but completely lacks a D-loop arm found in the canonical tRNA cloverleaf structure. In addition, the interaction of METTL8 with SARS2 could stimulate the aminoacyl transferase activity of SARS2 on mitochondrial tRNA-Ser isoacceptors. Future studies using reconstitution of the METTL8–SARS2 complex *in vitro* could shed light on the role of SARS2 with METTL8 as well as the mechanism of tRNA recognition.

Intriguingly, METTL8 appears to have arisen within the vertebrate lineage (24, 29, 30). Thus, METTL8 could have evolved distinct biological roles from the other Trm140 counterparts. Indeed, prior studies have linked METTL8 to adipogenesis as well as mouse embryonic stem cell differentiation (51, 52, 53). Moreover, METTL8 has also been found in a nucleolar RNA-binding complex that affects R-loop stability in genomic regions (34). However, the molecular role of METTL8 in these biological processes remains enigmatic. Our work suggests that METTL8 could play a key role in these diverse biological processes through the methylation of mt-tRNAs and subsequent impact on mitochondrial physiology.

While our article was in revision, an independent work on METTL8 was recently published (54). Both our study and the work by Schöller *et al*. use METTL8-deficient human cells to demonstrate that METTL8 is required for m^3^C formation in mt-tRNA-Ser-UGA and tRNA-Thr-AGU. Moreover, our study and Schöller *et al*. use biochemical reconstitution to demonstrate that METTL8 exhibits methyltransferase activity on tRNA substrates *in vitro*. In particular, we have shown that purified METTL8 can methylate mt-tRNA-Thr-UGU while Schöller *et al*. have found that purified METTL8 can methylate mt-tRNA-Ser-UGA. Thus, our study and the work of Schöller *et al*. provide independent corroboration of each other’s main conclusion that METTL8 is the mitochondrial enzyme catalyzing m^3^C formation in mt-tRNA-Ser-UGA and mt-tRNA-Thr-UGU.

Using mitochondrial ribosome profiling, Schöller *et al*. have found that METTL8-deficient cells exhibit an enrichment of codons in the P-site of mitochondrial ribosomes that are decoded by mt-tRNA-Ser-UGA, but not mt-tRNA-Thr-AGU. This result is consistent with our finding that the folding of mt-tRNA-Ser-UGA, but not mt-tRNA-Thr-AGU, is impacted by METTL8-deficiency. Together, our study and the work by Schöller *et al*. suggest that METTL8-dependent m^3^C formation is required for the proper folding and function of mt-tRNA-Ser-UGA in mitochondrial translation.

Similar to our findings in human HAP1 cells, Schöller *et al*. have observed that loss of METTL8 in human 293T cells has negligible impact on cell proliferation. However, Schöller *et al*. have found that knockout of METTL8 in a pancreatic cancer cell line can reduce cell growth. This finding is consistent with prior studies that have identified frameshift mutations in METTL8 in colorectal cancers and upregulation of METTL8 in breast cancer, an observation further documented by Schöller *et al*. in many types of cancers (55, 56). Moreover, Schöller *et al*. have found that METTL8-deficient human cells exhibit reduced respiratory chain activity and respiratory complex assembly. Thus, the proper maintenance of mitochondrial tRNA folding by METTL8-dependent modification could be important for cancer cell metabolism and homeostasis. Collectively, our study and the work by Schöller *et al*. provide a complementary framework for understanding the impact of METTL8-catalyzed tRNA methylation in translation and cellular physiology that could be a possible target for anticancer therapeutics.

## Experimental procedures

### Plasmids

The open reading frame (ORF) for METTL8 was RT-PCR amplified from HeLa human cervical carcinoma cell cDNA, cloned by restriction digest, and verified by Sanger sequencing. The cDNA clones for METTL8 correspond to NM_001321154.2. METTL8 without the N-terminal MTS sequence was PCR amplified and cloned from the ORF of METTL8. Primers used are listed in Table S1. The ORF for METTL8 and ΔMTS was cloned into either pcDNA3.1-Cterm-TWIN-Strep or pcDNA3.1-Cterm-GFP. The ORF for SARS2 was PCR amplified from cDNA plasmid HsCD00337767 (Plasmid Repository, Harvard Medical School) and cloned into pcDNA3.1-Cterm-3XFLAG.

For lentiviral expression constructs, Strep tagged-METTL8 and ΔMTS was first cloned into the pENTR.CMV.ON plasmid using NheI and NotI restriction sites. The entry vectors were recombined *via* LR clonase reaction (ThermoFisher) into the pLKO.DEST.puro destination vector to allow for stable integration of the target genes into human HAP1 cell lines.

### Mammalian tissue culture and CRISPR cell lines

The 293T human embryonic cell line was originally obtained from ATCC (CRL-3216). The HAP1 Human Male Chronic Myelogenous Leukemia cell line was obtained from Horizon Discovery Life Sciences. 293T human embryonic kidney cell lines were cultured in Dulbecco’s Minimal Essential Medium (DMEM) supplemented with 10% fetal bovine serum, 1X penicillin and streptomycin (ThermoFisher), and 1X Glutamax (Gibco) at 37 °C with 5% CO2. Cells were maintained and passaged every 3 days using 0.25% Trypsin. Human HAP1 AAVS WT and METTL8 KO cell lines were generated by CRISPR/Cas9 mutagenesis. Human HAP1 cell lines were cultured in Iscove’s Modified Dulbecco’s Medium supplemented with 10% fetal bovine serum and 1X penicillin and streptomycin at 37 °C with 5% CO2. Cells were passaged every 3 days with 0.05% Trypsin. For cell proliferation assays, 1 × 10^5^ cells were seeded into a 6-well plate. After 24, 48, 72, and 96 h, cells were trypsinized and counted using a Muse Flow cytometer. To assay mitochondrial membrane potential, we used the Muse mitopotential assay kit (Luminex). Another cell aliquot was treated with carbonyl cyanide 4-(trifluoromethoxy)phenylhydrazoneFCCP as a negative control, for 5 min before analysis.

For generation of stable cell lines, 2.5 × 10^5^ 293T cells were seeded onto 60 × 15 mm tissue culture dishes. 1.25 μg of pLKO.DEST.puro plasmids containing the cloned ORF of METTL8-TWIN, ΔMTS-TWIN, or empty vector along with a lentiviral packaging cocktail containing 0.75 μg of psPAX2 packaging plasmid and 0.5 μg of pMD2.G envelope plasmid was transfected into HEK293T cells using calcium phosphate transfection. Media was changed 16 h post-transfection. Forty eight and seventy two hours after transfection, the media containing virus was collected and filter sterilized through 0.45 μm filters. One milliliter aliquots were flash frozen for later infection.

For lentiviral infection in 293T or HAP1 cell lines, 2.5 × 10^5^ cells were seeded in 6-well plates. Twenty four hours after initial seeding, 1 ml of either virus or media for mock infection along with 2 ml of media supplemented with 10 μg/ml of polybrene was added to each well. The cells were washed with PBS and fed fresh media 24 h postinfection. Puromycin selection began 48 h after infection at a concentration of 2 μg/ml. Fresh media supplemented with puromycin was added every other day and continued until the mock infection had no observable living cells. Proper integration and expression of each construct was verified *via* immunoblotting.

### Subcellular localization

The prediction of an N-terminal mitochondrial presequence, cleavable localization signal, and TOM20 import signal was performed using MitoFates: http://mitf.cbrc.jp/MitoFates/cgi-bin/top.cgi) (35).

For localization of METTL8 tagged with GFP, 293T human cells were seeded onto coverslips in a 6-well plate followed by transfection with pcDNA3.1-METTL8-EGFP or pcDNA3.1-METTL8ΔMTS-EGFP using Lipofectamine 3000 reagent (Thermo Fisher). For mitochondrial localization, the cells were infected with baculovirus expressing RFP targeted to mitochondria (CellLight Mitochondria-RFP, BacMam 2.0, Life Technologies). To visualize the nucleus, Hoechst’s dye was added to the media for 30 min before the cells were washed with PBS, fixed with 4% formaldehyde, and mounted in Aqua Poly/Mount (Polysciences Inc) followed by imaging on a Nikon A1R HD.

### LC-MS analysis

Vector control or METTL8-Strep stably integrated 293T cell lines were seeded on two 150 × 25 mm tissue culture dish plates and grown until 70% confluency before the cells of each line were combined and harvested for protein extraction. Cell pellets were resuspended in 1 ml of hypotonic lysis buffer (20 mM Hepes pH 7.9, 2 mM MgCl_2_, 0.2 mM EGTA, 10% glycerol, 0.1 mM PMSF, 1 mM DTT) per 150 × 25 mm tissue culture plate. Cells were kept on ice for 5 min and then underwent a freeze-thaw cycle three times to ensure proper detergent-independent cell lysis. NaCl was then added to the extracts at a concentration of 0.4 M along with NP-40 to a final concentration of 0.2% and subsequently incubated on ice for 5 min and spun down at 14,000*g* for 15 min at 4 °C. In all, 1 ml of hypotonic lysis buffer supplemented with 0.2% NP-40 was added to 1 ml of the supernatant extract.

Strep tagged proteins were then purified by incubating whole cell lysates with MagStrep “type3” XT beads (IBA Life Sciences) for 5-6 h at 4 °C. Magnetic resin was washed three times in 20 mM Hepes pH 7.9, 2 mM MgCl2, 0.2 mM EGTA, 10% glycerol, 0.1% NP-40, 0.2 M NaCl, 0.1 mM PMSF, and 1 mM DTT. Proteins were eluted with 2X Buffer BX (IBA LifeSciences), which contains 10 mM D-biotin. To ensure all protein was efficiently eluted off the magnetic resin, the beads were left rotating in Buffer BX overnight at 4 °C. Two one-hour elutions were completed the following day, and all elutions were pooled together for each individual sample. The total eluate was then placed on a Spin-X UF 500 μl centrifugal concentrator (Corning) and spun at 15,000*g* for approximately 1 h and 15 min at 4 °C.

For protein separation, 15 μl of concentrated eluate was fractionated on a NuPAGE 4 to 12% Bis–Tris Protein gel (ThermoFisher). The gel was fixed overnight in 40% ethanol and 10% acetic acid. The gel was incubated in sensitizing solution (30% ethanol, 0.2% sodium thiosulphate, and 6.8% sodium acetate) for 30 min before being washed three times with water for 5 min each wash. The gel was then stained in 0.25% silver nitrate for 20 min and washed twice more with water for 1 min each time. The bands were visualized by developing in 2.5% sodium carbonate and 0.015% formaldehyde and allowed to incubate until bands appeared. The remainder of each eluate (∼65 μl) was loaded on a NuPAGE 4 to 12% Bis–Tris protein gel and briefly fractionated to yield a single gel band corresponding to the majority of proteins within each purification. The gel bands were excised using a razor blade and subject to in gel reduction, alkylation, and trypsin digest by the URMC Mass Spectrometry Resource Lab.

Peptides were injected onto a homemade 30 cm C18 column with 1.8 μm beads (Sepax), with an Easy nLC-1000 HPLC (Thermo Fisher), connected to a Q Exactive Plus mass spectrometer (Thermo Fisher). Solvent A: 0.1% formic acid in water and solvent B: 0.1% formic acid in acetonitrile. The gradient began at 3% B and held for 2 min, increased to 30% B over 13 min, increased to 70% over 2 min and held for 3 min, then returned to 3% B in 2 min and re-equilibrated for 8 min, for a total run time of 30 min. For peptides isolated from the single gel band corresponding to the majority of proteins, the gradient began at 3% B and held for 2 min, increased to 30% B over 41 min, increased to 70% over 3 min and held for 4 min, then returned to 3% B in 2 min and re-equilibrated for 8 min, for a total run time of 60 min. The Q Exactive Plus was operated in data-dependent mode, with a full MS1 scan followed by eight data-dependent MS2 scans. The full scan was done over a range of 400 to 1400 m/z, with a resolution of 70,000 at m/z of 200, an AGC target of 1e6, and a maximum injection time of 50 ms. The MS2 scans were performed at 17,500 resolution, with an AGC target of 1e5 and a maximum injection time of 250 ms. The isolation width was 1.5 m/z, with an offset of 0.3 m/z and a normalized collision energy of 27 was used.

Raw data was searched using the Mascot search engine (Matrix Science) within the Proteome Discoverer software platform, version 1.4 (Thermo Fisher), using the SwissProt human database that was downloaded in December of 2015 with a total of 20,541 entries. Trypsin was selected as the enzyme allowing up to two missed cleavages, with an MS1 mass tolerance of 10 ppm and an MS2 mass tolerance of 25 mmu. Carbamidomethyl was set as a fixed modification, while oxidation of methionine was set as a variable modification. Percolator was used as the FDR calculator, filtering out peptides which had a q-value greater than 0.01 (57). The Mascot scores of individual peptides were calculated as the absolute probability that the observed peptide match is a random event when matching spectra to all the expected spectra of a given proteome (58). The Mascot score of a given peptide is equal to −10 × Log_10_(P), where P is the absolute probability. The Mascot scores for individual proteins were then calculated based upon the summation of the individual peptides for all peptides matching a given protein. The LC-MS analysis was performed once with each sample. The mass spectrometry proteomics data have been deposited to the ProteomeXchange Consortium *via* the PRIDE partner repository (59) with the dataset identifier PXD030418.

### Transient transfections and Protein–RNA purifications

293T cells were transfected *via* calcium phosphate transfection method (60). Briefly, 2.5 ×10^6^ cells were seeded on 100 × 20 mm tissue culture grade plates (Corning) followed by transfection with 10 to 20 μg of plasmid DNA. Media was exchanged 24 h after transfection. Cells were harvested 48 h post transfection by incubation in 0.25% trypsin and neutralization with media, followed by centrifugation of the cells at 700×*g* for 5 min followed by subsequent PBS wash and a second centrifugation step.

Protein was extracted by the hypotonic lysis protocol immediately after the cells were harvested post-transfection as was outlined in the above section. 0.5 ml of hypotonic lysis buffer was added to each sample per 100 × 200 mm tissue culture plate, with an additional 0.5 ml of hypotonic lysis buffer supplemented with NP-40 added for a final total of 1 ml of extract. Each extract was split into two 0.5 ml aliquots and flash frozen for later use. TWIN-Strep tagged proteins were then purified by incubating whole cell lysates from the transiently-transfected cell lines (0.5 ml) with 50 μl of MagStrep “type3” XT beads (IBA Life Sciences) for 2 to 4 h at 4 °C. Magnetic resin was washed three times in 20 mM Hepes pH 7.9, 2 mM MgCl_2_, 0.2 mM EGTA, 10% glycerol, 0.1% NP-40, 0.2 M NaCl, 0.1 mM PMSF, and 1 mM DTT. Proteins were eluted with 1X Buffer BX (IBA LifeSciences). Purified proteins were visualized on a BOLT 4 to 12% Bis–Tris Plus gel (Life Technologies) and then transferred to Immobilon-FL Hydrophobic PVDF Transfer Membrane (Millipore Sigma) with subsequent immunoblotting against either the FLAG-epitope tag or TWIN-Strep epitope tag (Anti-FLAG M2, Sigma-Aldrich; THE NWSHPQFEK antibody, GenScript).

We also utilized this purification procedure followed by TRIzol RNA extraction directly on the beads to identify copurifying RNA with each protein of interest. Beads first underwent three washes in the lysis buffer as mentioned previously and then resuspended in 250 μl of molecular biology grade RNAse-free water (Corning). Ten microliters of the bead-water mixture was taken for immunoblotting analysis where the beads were mixed with 2X Laemmeli sample buffer (Bio-Rad) supplemented with DTT and boiled at 95 °C for 5 min prior to loading onto a BOLT 4 to 12% Bis–Tris Plus gel (Life Technologies). RNA extraction followed the TRIzol LS RNA extraction protocol (Invitrogen). RNA was resuspended in 5 μl of RNAse-free water and loaded onto a 10% polyacrylamide, 7M urea gel. The gel was then stained with SYBR gold nucleic acid stain (Invitrogen) to visualize RNA followed by transferring onto an Amersham Hybond-XL membrane and subsequent Northern blot procedures outlined below.

### CRISPR gene editing

CRISPR-Cas9 constructs were generated by cloning double-strand oligonucleotide inserts into pX333 (Addgene). Sequence guide RNAs (sgRNAs) were designed to target exon 3 of the METTL8 ORF (Table S1) and span a region of 72 bp. sgRNAs were also designed to target the AAVS ORF and act as our WT control. HAP1 human cells were seeded at 2.5 × 10^5^ in a 6-well plate and the next day transfected with px333-sgRNA4-sgRNA-5 using Lipofectamine 3000 (ThermoFisher). Single cell clones were isolated and placed into four 96-well plates. Single cell clones were allowed to grow until a single colony was visible. Media was replaced at the end of each week spanning around 3 weeks. Each single colony was trypsinized with 0.05% trypsin and neutralized with media to reseed in a single well of a 24-well plate and allowed again to grow. Once the wells reached ∼70% confluency, cells were either harvested for DNA extraction (Qiagen) or frozen down for long-term storage. The presence of CRISPR-induced mutations in the *METTL8* gene was detected by PCR amplification and confirmed by Sanger sequencing using primers listed in Table S1.

### Immunoblot assays

To verify the loss of METTL8, cell extracts were loaded onto BOLT 4 to 12% Bis–Tris gels (ThermoFisher) followed by immunoblotting onto PVDF membrane and probing with METTL8 antibody (ThermoFisher, cat. No PA557265, 1:500 dilution) and anti-Actin C4 (EMD Millipore, cat. No MAB1501, 1:000 dilution). Expression of METTL8 in stably infected cell lines were characterized by immunoblotting with the anti-Strep antibody (THE NWSHPQFEK antibody, Genscript, cat. No. A01732, 1:1000 dilution). Transient transfections of TWIN-Strep and FLAG-tagged proteins were also loaded onto BOLT 4 to 12% Bis–Tris gels, immunoblotted to PVDF membranes, and probed with anti-TWIN-Strep (THE NWSHPQFEK antibody, Genscript, cat. No. A01732, 1:1000 dilution), anti-FLAG M2 (anti-FLAG M2, Sigma-Aldrich, 1:3000 dilution), or anti-SARS2 (Abclonal, A12297, 1:1000 dilution). Image analysis of immunoblots was performed using Image Studio software (Li-Cor).

### RNA analysis

3-methylcytidine modification status was monitored using the Northern blot-based PHA assay. To conduct the PHA assay, probes were designed to hybridize upstream and downstream of residue 32 (oligos listed in Table S1). A total of 5 μg of RNA was loaded onto a 10% polyacrylamide, 1xTBE, 7 M urea gel, and transferred onto an Amersham Hybond-XL membrane (GE Healthcare) for Northern blotting analysis. Oligonucleotides used to detect RNAs are listed in Table S1. The oligos were radiolabeled by T4 polynucleotide kinase (NEB) with adenosine [γ^32^P]-triphosphate (6000 Ci/mmol, Amersham Biosciences) following standard procedures. Northern blots were visualized by phosphor-imager analysis and stripped *via* two incubations at 80 °C for 40 min in a buffer containing 0.15 M NaCl, 0.015 m Na-citrate, and 0.1% SDS. Image analysis of Phosphorimager scans were performed using ImageJ open-source software.

To monitor m^3^C formation in mt-tRNA-Thr-UGU by primer extension analysis, we pre-annealed 0.625 pmol of 5′-^32^P radiolabeled DNA oligo to either 3.2 μg of extracted total RNA or 100 ng of *in vitro* transcribed tRNA from the methyltransferase assays. The pre-annealed oligo and RNA mixture was mixed with 1.4 μl 5X hybridization buffer (250 mM Tris, pH 8.5 and 300 mM NaCl) to a total of 7 μl. The mixture was then heated to 95 °C for 3 min and slow cooled to 42 °C. The reaction was mixed with 7 μl of extension mix (0.12 μl of AMV Reverse Transcriptase (Promega), 1.4 μl 5X AMV buffer, 0.56 μl of 1 mM dNTPs, and 4.92 μl of RNase-free water). The reaction was incubated at 42 °C for 1 h before being mixed with 2X formamide loading dye, heated at 95 °C for 3 min, and run on a 7M urea-1X TBE-18% polyacrylamide gel. Gels were exposed to a phosphor screen (GE Healthcare) for 1 h and scanned *via* Phosphorimaging. Image analysis of Phosphorimager scans were performed using ImageJ open-source software.

For analysis of RNA structure under native conditions, 3 μg of RNA was resuspended in 6x loading dye (NEB Biolabs, cat. No B7024S) and separated on a 20% PA gel without urea run at 160 V at 4 °C. The RNA samples were kept on ice until loaded on the gel. RNA was then transferred onto an Amersham Hybond-XL membrane and subsequent probing with radiolabeled oligonucleotides as described above.

### *In vitro* methyltransferase assay

The mt-tRNA-Thr-UGU template was created by *in vitro* transcription and all relevant DNA primers are located in Table S1. A double-stranded template including a T7 promoter upstream of the tRNA gene sequence was ordered from IDT as a gBlock DNA fragment and then PCR amplified using a T7 promoter forward primer and a reverse primer complementary to the 3′ end of mt-Thr-UGU. PCR amplification was done using Herculase II Fusion DNA polymerase following standard protocol. The PCR parameters were 95 °C for 2 min followed by 35 cycles of 95 °C for 20 s, 49 °C for 20 s, and 72 °C for 30 s, ending with 72 °C for 2 min. The PCR product was run on a 2% agarose gel and the bands were excised and DNA was purified using a Qiagen Gel Extraction kit. *In vitro* transcription was done using Optizyme T7 RNA polymerase (ThermoFisher) following standard protocol, incubated at 37 °C for 3 h. The reaction was then treated with DNase (RQ1 DNase, Promega) at 37 °C for 30 min. RNA was purified and concentrated using an RNA Clean and Concentrator Zymo-Spin Column (Zymo Research). Integrity of our tRNA was visualized on a denaturing 7M urea-1X TBE-15% polyacrylamide gel.

Vector control and METTL8 was transiently expressed and purified as described above. Protein expression and purification was verified through immunoblotting as shown in Figure 3*D*. Prior to incubation with purified protein, the *in vitro* transcribed tRNA was refolded by initial denaturation in 5 mM Tris pH 7.5, 0.16 mM EDTA, and heated to 95 °C for 2 min followed by incubation on ice for 2 min. Refolding of the tRNA was done at 37 °C for 20 min in Hepes pH 7.5, MgCl_2_, and NaCl. For the methyltransferase assay, 100 ng of refolded tRNA was incubated with ∼200 nM of purified METTL8 in 50 mM Tris pH 7.5, 0.1 mM EDTA, 1 mM DTT, and 0.5 mM *S-*adenosylmethionine (NEB) for 4 h at 30 °C. RNA was purified using TRIzol as denoted above. Formation of m3C was monitored *via* primer extension analysis.

### CRISPRi

Lentiviral CRISPRi constructs were generated by cloning double-strand oligonucleotide inserts into pLV hU6-sgRNA hUbC-dCas9-KRAB-T2a-Puro (Addgene, 71236). The oligonucleotides are listed in Table S1. Lentiviral constructs were transfected with packaging plasmids (psPAX2 and pMD2.G; Addgene) into 293T cells for lentivirus production. The 293T cell line was subsequently infected with lentivirus in the presence of hexadimethrine bromide followed by selection with puromycin. Knockdown was measured by immunoblot with an anti-SARS2 antibody (Abclonal, A12297).

## Data availability

The mass spectrometry proteomics data have been deposited to the ProteomeXchange Consortium *via* the PRIDE partner repository (59) with the dataset identifier PXD030418.

## Supporting information

This article contains supporting information.

## Conflict of interest

The authors declare that they have no conflicts of interest with the contents of this article.
