# Supplementary material for: Methyltransferase METTL8 is required for 3-methylcytosine modification in human mitochondrial tRNAs
Source: J Biol Chem. 2022 Mar 3;298(4):101788. doi: 10.1016/j.jbc.2022.101788 (PMC8980813; doi:10.1016/j.jbc.2022.101788)
Supplement: Supplementary Figures S1–S7 and Table S1 [file mmc1.docx]

**Methyltransferase METTL8 is required for 3-methylcytosine modification in human mitochondrial tRNAs**

Jenna M. Lentini, Rachel Bargabos, Chen Chen, and Dragony Fu*

**Figure S1.** METTL8 exhibits localization in mitochondria that is dependent upon a mitochondrial targeting signal (MTS).

**Figure S2.** METTL8 interacts with mt-tRNA-Ser and mt-tRNA-Thr.

**Figure S3.** Isogenic control-WT and METTL8-KO cells exhibit similar rates of proliferation and mitochondrial membrane potential as isogenic wildtype cells.

**Figure S4.** Northern blot analysis using the Positive Hybridization in the Absence of Modification (PHA) assay.

**Figure S5.** Immunoblot of the indicated human cell lines probed with anti-METTL8 antibody.

**Figure S6.** Analysis of m3C modification in SARS2-knockdown cell lines

**Figure S7.** The native migration pattern of mt-tRNA-Ser is altered in METTL8-KO cell lines.

**Table S1.** Table S1. List of primers and oligonucleotides used in this study.

**Figure S1.** METTL8 exhibits localization in mitochondria that is dependent upon a mitochondrial targeting signal (MTS). (A, B) Confocal microscopy images of 293T cells transiently transfected with constructs expressing METTL8-WT or METTL8-ΔMTS fusion proteins with GFP. Mitochondria were identified using mitochondrion-targeted red fluorescent protein and nuclear DNA was stained with Hoechst. Overlap of red mitochondria and green GFP signal is displayed by yellow merged color. (A) Images correspond to the full field-of-view of the images in Fig. 2C. (B) Images are from a different section of the slide.

**Figure S2.** METTL8 interacts with mt-tRNA-Ser and mt-tRNA-Thr. Repeat of METTL8 purification in Figure 4. (A) Immunoblot analysis of streptactin purifications from human cells expressing control, METTL8, or METTL8-ΔMTS fused to the twin-Strep tag. The immunoblot was probed with anti-TwinStrep and anti-actin antibodies. (B) Nucleic acid stain of RNAs extracted from the indicated input or purified samples after denaturing PAGE. The migration pattern of 5.8S rRNA (~150 nt), 5S rRNA (~120 nt) and tRNAs (~70–80 nt) are denoted. (C) Northern blot analysis of the gel in (B) using the indicated probes. Input represents 2% of total extracts used for purification. The percentage yield represents the amount of RNA in the Strep purification that was recovered from the total input. Quantification was performed on the blot shown.

**Figure S3.** Isogenic control-WT and METTL8-KO cells exhibit similar rates of proliferation and mitochondrial membrane potential as isogenic wildtype cells. (A) Cell number as a function of time in days for the indicated cell lines. (B) Percent of live cells with polarized mitochondria as measured by flow cytometry. Cells were treated with FCCP as a positive control for dissipation of the mitochondrial membrane potential. Error bars represent the standard deviation of the mean.

**Figure S4.** Repeat of Figure 5E. Northern blot analysis using the Positive Hybridization in the Absence of Modification (PHA) assay with probes designed to detect m^3^C at position 32 and a control probe that hybridizes to a different area of the same tRNA.

**Figure S5.** Immunoblot of the indicated human cell lines probed with anti-METTL8 antibody. Load Control is a non-specific band located on this gel.


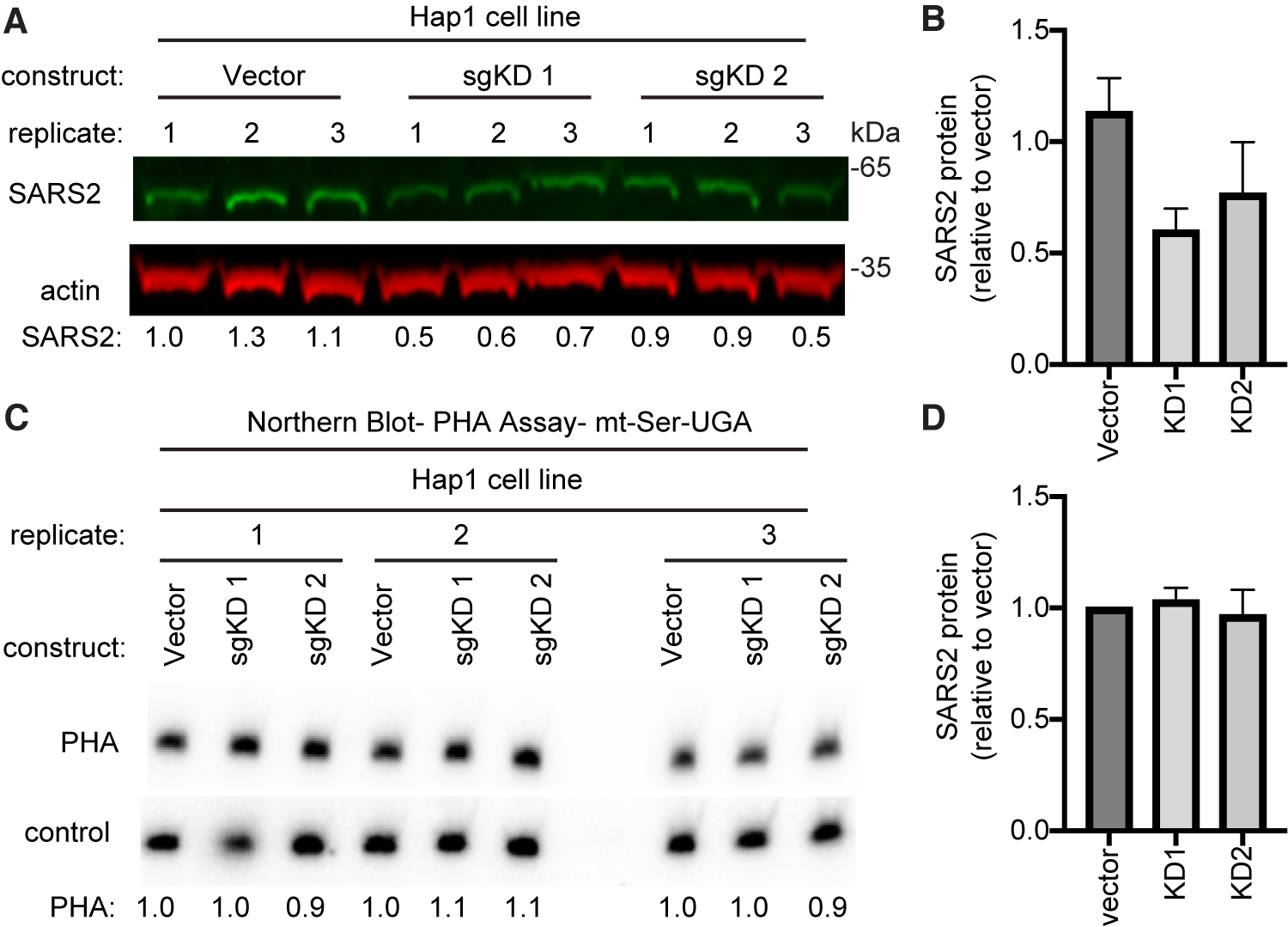


**Figure S6**. Depletion of SARS2 in 293T cells and analysis of m^3^C modification in mt-tRNAs. (A) Immunoblot analysis of SARS2 expression in Human Hap1 cell lines containing the indicated lentiviral CRISPRi constructs. Actin was used as a loading control. (B) Quantification of the replicate sample loadings for SARS2 in (A). (C) Northern blot analysis using the Positive Hybridization in the Absence of Modification (PHA) assay with probes designed to detect m^3^C at position 32 and a control probe that hybridizes to a different area of the same tRNA. (D) Quantification of PHA blots. PHA quantification represents the ratio of PHA versus control probe signal expressed relative to the vector cell line.

**Figure S7.** Independent replicate of Figure 7. The native migration pattern of mt-tRNA-Ser is altered in METTL8-KO cell lines. (A) Total RNA from the indicated cell lines was fractionated on non-denaturing gels followed by transfer and hybridization with probes against the indicated tRNAs. The predominant band for each tRNA species is denoted by the arrowhead. The slower migrating mt-tRNA-Ser species found in METTL8-KO cell lines is denoted by the arrow. (B) Native gel analysis of mt-tRNA-Ser and Thr from the indicated Rescue cell lines. The predominant band for each tRNA species is denoted by the arrowhead and the slower migrating mt-tRNA-Ser species is denoted by the arrow.

| **Primer Name** | **Sequence 5’🡪3’** |
| --- | --- |
| METTL8-F-HindIII | GAACT AAGCTT ATGAATATGATTTGGAGAAATTCCATTTCTTG |
| Mettl8 R-C term | CATGA GCGGCCGC GTCTTGTGAAAGGAGTGTAGATACCATATTG |
| DELTAMTS METTL8 HINDIII F | GAACTAAGCTTATGCAAAGTGGTTACCACCCAGTGG |
| SARS2 F-KPNI | AAAGGTACC ATGGCTGCGTCCATGG |
| SARS2 R-NOTI | CATGA GCGGCCGC GCTTACAGCAGGCTGGCCAGGC |
| METTL8 sgRNA #4 F | CACCG GGATCACATGCAGTGGTCTA |
| METTL8 sgRNA #4 R | AAAC TAGACCACTGCATGTGATCC C |
| METTL8 sgRNA #5 F | CACCG CGAGTCCTTCTGGAAGAGCA |
| METTL8 sgRNA #5 R | AAAC TGCTCTTCCAGAAGGACTCG C |
| AAVS sgRNA F | CACCGGGGGCCACTAGGGACAG |
| AAVS sgRNA R | AAACATCCTGTCCCTAGTGGCCCCC |
| METTL8 KOs PCR and Seq F | GCAGCCGTTTCCCAGAGGTAG |
| METTL8 KOs PCR and Seq R | TGGGAATTCTACCAAATGGAAC |
| mt-Ser-UGN PHA nt 19-42 | CAG ACT GGT TTC AAG CCA ATC CCA |
| mt-Ser-UGN Control nt 43-63 | AGGAGGGAATCGAACCCCCC |
| mt-Thr-UGU PHA nt 20-38 | GTTTACAAGACTGGTGTAT |
| mt-Thr-UGU Control nt 41-61 | TTGGAAAAAGGTTTTCATCTC |
| mt-Ile GAU nt 30-54 | TTAAGCTCCTATTATTTACTCTATC |
| Cyto-Thr-AGU PHA nt 25-42 | TGTTTACTAGACAGGCGC |
| Cyto-Thr-AGU Cont nt 41-60 | TTCGAACCCAGGATCTCCTG |
| Cyto- Ser-UGA PHA nt 23-42 | TGGATTTCAAGTCCATCGCC |
| Cyto-Ser-UGA Cont nt 42-64 | AACCTGCGCGGGGAAACCCCAAT |
| Cyto-Arg CCU PHA nt 25-40 | GCTTAGGAGGCCAATG |
| Cyto-Arg CCU Cont nt 40-62 | GGACTCGAACCCACAaTCCCTGG |
| CRISPRi-SARS2-F1 | GTTT G AATTGGTCTAAACGCGGAGT |
| CRISPRi-SARS2-R1 | AAAC ACTCCGCGTTTAGACCAATT C |
| CRISPRi-SARS2-F3 | CACC G GGCGACGAAGGACTCTATCG |
| CRISPRi-SARS2-R3 | AAAC CGATAGAGTCCTTCGTCGCC C |
| mt-Thr-UGU template | GCATATGGAGAATTTGTA CGC GGATCC GAAT TAATACGACTCACTATA GTCCTTGTAGTATAAACTAATACACCAGTCTTGTAAACCGGAGATGAAAACCTTTTTCCAAGGACA AAGCTTGAACT |
| mt-Thr-UGU Reverse primer | TGTCCTTGGAAAAAGGTTTTC |
| T7 forward primer | GAATTAATACGACTCACTATAG |
| mt-Thr-UGU primer extension primer | AAGGTTTTCATCTCCGGTT |

**Table S1.** List of primers and oligonucleotides used in this study.
